# A comparison of using online market panel members and traditional methods in control recruitment for a case–control study during a national outbreak of STEC O145, United Kingdom

**DOI:** 10.1017/S0950268826101599

**Published:** 2026-05-19

**Authors:** Grace King, Carys Rees, Clare Sawyer, Ann Hoban, Thomas Inns, Orlagh Quinn, Yanshi, Amy Douglas, André Charlett, Lesley Larkin, Carmellie Inzoungou-Massanga, Motolani Awokoya, Maria Waghorn, Oluwakemi Olufon, Andrew Nelson, Christopher Williams, Paul Millar, Catriona Dobosz, Genna Leckenby, Caoimhe McKerr, Sema Nickbakhsh, Sooria Balasegaram

**Affiliations:** 1 UK Health Security Agency, London, UK; 2 Public Health Wales, Cardiff, UK; 3 Public Health Agency, Belfast, UK; 4 Public Health Scotland, Glasgow, UK

**Keywords:** Control Groups, Case-Control Studies, Disease Outbreaks, Gastrointestinal Diseases, Methods

## Abstract

National foodborne outbreaks of gastrointestinal disease often require rapid case–control investigations to identify the source. Online market panels offer a potential alternative to traditional control recruitment. We compared market panel controls to traditional controls in case–control recruitment during a 2024 UK outbreak of STEC O145. Two case–control studies were conducted for two different control groups (a) *Salmonella* cases as case–controls and (b) online market panel members. Timeliness, cost, and resources were compared, and a logistic regression compared findings in a control–control analysis. In total, 43 cases of STEC O145, 63 *Salmonella* case–controls, and 93 panel controls were recruited. Neither control group reached the recruitment target for the younger adult age group. *Salmonella* case–controls had a ninefold greater staff time to recruit and cost five times more than panel controls (£25.82 vs. £4.99 per control), partially due to interviewer-administered questionnaires compared with self-completion by panel controls. Both analytical approaches identified the same outbreak source, with no significant differences in exposures between the control groups. The cost and resource savings associated with panel controls justify their use as a standard procedure in outbreak investigations. We recommend exploring engagement with age groups that are difficult to recruit and assessing alternative strategies to reach them.

## Introduction

Foodborne gastrointestinal disease outbreaks can be geographically widespread, occur rapidly in time, and cause severe outcomes for individuals affected. Shiga toxin-producing *Escherichia coli* (STEC) is a food-borne gastrointestinal pathogen of significant public health concern due to the potential for severe morbidity and mortality in people infected [1]. In the UK, historically, large outbreaks with links to fresh produce have been caused by serotype O157 [2–4]. In 2024, a UK-wide outbreak of STEC O145 was identified, the largest outbreak since the introduction of routine whole genome sequencing (WGS) [5].

A traditional method of epidemiological investigation to identify exposures associated with an infection or health condition is case–control studies, where people with the infection or health condition are compared to people who do not have the infection or health condition [6]. National foodborne outbreaks of gastrointestinal disease require rapid implementation of public health control measures to prevent further spread of disease with potentially severe outcomes.

Online market panel members are being increasingly used as controls for case–control studies for the investigation of gastrointestinal disease outbreaks [7]. Market panels offer monetary reward for the completion of an online questionnaire emailed to individuals signed up to the service. Traditional methods of recruiting population controls, which involve selecting a sample from the population at risk that does not have the disease under investigation, for example, telephoning cases with a different infection or using random digit dialling, are often more resource-intensive, costly, and time-consuming, which delays public health action [8, 9].

Given the infrequent but increasing use of market panels in the investigation of GI outbreaks in the UK, it is useful to consider whether they still are an appropriate alternative to traditional epidemiological methods of control recruitment. Between May and July 2024, 293 cases of STEC O145:H28 *stx2a/eae* belonging to the same five single-nucleotide polymorphism (SNP) single linkage cluster were identified across the UK [5]. The epidemiological, food-chain, and environmental investigations identified pre-packaged sandwiches containing lettuce as the likely source of the outbreak. Both methods of control recruitment were used in case–control studies responding to this national outbreak of STEC O145 to identify the infection source. The timeliness, cost, and resources required for both methods of control recruitment in this outbreak were compared, in addition to comparing the demographics and exposures of the two control groups and the findings from the analytical studies.

## Methods

In May 2024, an outbreak control team (OCT) was convened to identify the cause of the UK-wide outbreak of STEC O145:H28 *stx2a/eae.* Hypothesis generation in the form of a case–case study compared exposures reported by English cases in the outbreak cluster with other English STEC cases as controls using data from enhanced surveillance questionnaires (ESQ) stored in UKHSA’s National Enhanced Surveillance System for STEC (NESSS). Following hypothesis generation, an analytical study using targeted questionnaires with more detail on significant exposures, including pre-packaged sandwiches, eating out, and salad consumption, was conducted using two sets of controls [5].

### Recruitment of controls from a market research panel

Market panel controls (MPCs) were recruited through Company X. Company X has more than 200,000 panellists in the UK. MPCs were frequency matched to cases based on country of residence in the UK and age groups with three strata (11–18 years age group, 19–34 years age group, and 35–70 years age group). For the strata age 11–18 years old, parents were asked to complete a survey on behalf of their child. For each age strata, company X contacted a randomly selected group of panel members, who were resident in areas of the UK where cases resided, to complete the online survey for a reward. Exclusion criteria were reported foreign travel, experience of GI symptoms in the 7 days before completing the survey, or being outside the age boundaries for control recruitment. Individuals were asked about their food history in the 7 days prior to completing the survey.

### Recruitment of controls from salmonella cases

Cases of salmonellosis reported in the time frame of interest and resident in the countries within the UK, were contacted by their respective public health agency based on residence for control recruitment. The salmonellosis cases were excluded using a similar exclusion criterion as for the MPCs. The exclusion criteria, which excluded MPCs who experienced GI symptoms in the previous 7 days, did not apply, but salmonellosis cases were additionally excluded as controls if they were part of an ongoing outbreak investigation, to avoid multiple interviews/surveys. An ongoing outbreak investigation was defined as a salmonellosis case in a WGS-linked five SNP cluster, which was under investigation by the public health agency undertaking the questionnaire, with an Incident Management Team (IMT). *Salmonella* case–controls were recruited from all countries within the UK (England, Scotland, Wales, and Northern Ireland). Individuals were asked about their food history in the 7 days prior to symptom onset. Due to the age distribution of salmonellosis cases identified, the age stratification for the adult age groups was amended to 19–29 years and 30–70 years. The *Salmonella* case–controls were interviewed by telephone by a group of investigators in the respective agency. The interviewer used an online questionnaire to document the answers from the respondent. If the *Salmonella* case–control failed to answer the phone, a survey link was sent via email or text message for self-completion. The interview questions were similar to those received by the MPCs in the online questionnaire, with the removal of the question about experiencing GI symptoms in the previous 7 days. *Salmonella* case–controls resident in Wales were contacted via email from Public Health Wales with a survey link for self-completion in English or Welsh.

### Timeliness, resources, and cost associated with control recruitment

The time, average cost per control, and number of staff required were summarised and compared.

Time for MPCs was described for the end-to-end processes, from contacting Company X to closing the survey, and from launching the survey to closing the survey. For *Salmonella* case–controls, this was the period from when interviews began until the decision was made to end recruitment. Survey design was not included in time.

Cost per control was calculated for the MPCs by dividing the procurement costs billed from Company X by the number of people who successfully completed the survey. For the *Salmonella* case–controls, the cost per control was calculated by taking into account the following factors: the time taken to complete a full interview with data entry, the time spent contacting people who declined to take part or did not respond to the call, and the hourly rate for the minimum pay band of the staff member contacting the individuals. The time taken to contact controls and complete interviews was estimated by the interviewers who undertook the interview and entered the details into an online survey platform. The estimation was 30 min for the completed interviews and 10 min for contacting people who were non-respondents.

### Comparison of control groups

The findings from the analytical outbreak case–control studies, which looked for associations with food exposures and illness, were compared. The analytical methods undertaken in the case–control studies used in the outbreak investigation have been previously described [5].

An independent groups t-test was used to test for differences in mean age, and a Pearson’s Chi-squared test was used to test for differences in sex and country of residence between the control groups.

A univariable control–control analysis, comparing exposures for the *Salmonella* case–controls to the MPCs, was conducted. The food exposure variables that were significantly associated with being in one of the control groups (*P* < 0.1) in the univariable analysis were included in a multivariable Firth logistic regression model [10]. Age and sex were included in the model as *a priori* potential confounding variables. A forward stepwise approach was used for model construction. A false discovery rate (FDR) procedure was conducted using the Benjamini–Hochberg method (using the R package ‘stats’ and ‘p.adjust’ function in R) to control the proportion of false-positive results in the single variable analysis when comparing exposures in the two control sets [10].

Analysis was undertaken in RStudio version 4.4.2 and STATA 18.

## Results

### Response rates

For the outbreak case–control study, 43 cases with STEC O145 infection who were part of the outbreak cluster completed telephone interviews and were included in the analysis.

A total of 224 *Salmonella* case–controls were identified as being within the age boundaries for control recruitment, with no foreign travel recorded on the laboratory referral form for the week prior to the individuals’ onset. Of these 224 eligible case–controls, 204 (91%) were contacted for interview. In total, 73 *Salmonella* case–controls completed the control questionnaires (36%); however, ten ended up being excluded due to foreign travel being reported by the individual in the week prior to their symptom onset. The overall response rate for *Salmonella* case recruitment was 31% (63/204). Over half (111, 54%) could not be contacted by telephone or failed to self-complete the questionnaire when contacted using alternative methods, 18 (9%) did not meet the eligibility criteria, and 12 (6%) declined to complete the survey. The number of complete responses from *Salmonella* case–controls was 74% of the initial target (*n* = 85) and only met the recruitment target for the youngest age-group stratum (11–18 years). The final ratio of *Salmonella* case–controls to STEC O145 was 1.5:1.

The number of complete responses from MPCs was 93, 89% of the initial target (*n* = 104). The strata of 19–34 years did not meet the target number of responses (37/57, 65%). The final ratio of MPCs to cases of STEC O145 was 2.2:1.

### Timeliness of recruitment

The recruitment of *Salmonella* case–controls (*n* = 63) took 3 days. The end-to-end process of recruiting MPCs (*n* = 93) took five working days, including 3 days for the survey provider to respond to the initial request. Following the response to the request, the survey went live after 7 hours. For two of the age-group strata, recruitment was completed within 1 day. It took a further 1 working day to finish recruitment for the final age group. The recruitment for the final age group ended prior to the recruitment target being reached due to the urgency for analysis to be undertaken. Excluding the initial response from the survey provider, recruitment of MPCs took two working days.

### Resource and cost required for recruitment

The recruitment of *Salmonella* case–controls (*n* = 63) involved 253 telephone calls, 46 emails, and 206 text messages. The total number of staff involved in identifying and following up *Salmonella* case–controls was nine. In Wales, two additional staff members were required to aid in the translation of the survey from English to Welsh and in entering the survey onto a different data collection platform. For liaison with Company X to distribute the survey and support data collection for the MPC’s, three staff members were involved.

An estimation of 30 min of staff time was needed to complete the full survey with the *Salmonella* case–controls over the telephone. For calls to people who declined to participate, were removed following the screening questions, or those who did not answer the call following a number of attempts, an average of ten minutes of staff time was required per control. An hour was required for control identification, and a further hour was used for translation of the survey from English into Welsh. In total, 62.5 hours of staff time were required to obtain the 63 *Salmonella* case–controls. On the other hand, for MPC recruitment, liaison with Company X involved 7 hours of staff time to ensure the survey went live.

Including resources required to identify controls and telephone controls who declined to participate, were screened out, or could not be contacted, the average cost to recruit each *Salmonella* case–control (*n* = 63) was £25.82. For the recruitment of MPCs, including the costs of project management, programming, hosting, and outputs, the total invoiced cost was £310.40. Including staff time to liaise with the provider, this total increased to £463.77, averaging out at £4.99 per control.

### Demographics of control groups

The median age for MPC’s was 33 (IQR: 26–55), and the median age for *Salmonella* case–controls was 31 (IQR: 20.5–55.5). A higher proportion of females was observed for both MPC’s (62%) and *Salmonella* case–controls (65%). Demographic characteristics were similar when comparing each control group with cases for age, sex, and country of residence.

The distribution of sex (X^2^(1, *N* = 156) = 0.03, *p* = 0.86), age (t(154) = 0.68, *p* = 0.50), and country of residence (X^2^(3, *N* = 156) = 4.3, *p* = 0.24) was also similar between the control groups. The *Salmonella* case–controls included controls from Northern Ireland; none were recruited from the MPCs.

### Findings of case–control studies

In the final multivariable models for both studies, the main findings for the two studies were the same (Table 1). Pre-packaged sandwiches containing lettuce were significantly more likely to be consumed by cases than either set of controls (*Salmonella* case–controls: aOR: 7.1, 95% CI: 2.3–21.5, *p* = 0.001; MPCs: aOR: 4.8, 95% CI: 1.9–12.0, *p* < 0.001).

**Table 1.** Final multivariable analysis of odds of infection with STEC O145 using Salmonella cases as case–controls (Model 1) and market panel controls (Model 2) Table 1. long description.

Model 1: STEC O145 cases with Salmonella cases as case–controls							
Exposure	Cases (*n* = 43)		Controls (*n* = 63)		aOR	95% CI	*p* value
	*n*	%	*n*	%			
Age (ref: 11–18 year olds)							
Age: 11–18 year olds	6	14.0	12	19.0	REF		
Age: 19–29 year olds	25	58.1	18	28.6	4.4	0.9–21.3	0.063
Age: 30–70 year olds	12	27.9	33	52.4	2.3	0.5–10.8	0.300
Sex (ref: female)							
Female	28	65.1	41	65.1	REF		
Male	15	34.9	22	34.9	1.2	0.4–3.1	0.280
Consumption of any lettuce leaf in a pre-packaged sandwich	25	58.1	6	9.5	7.1	2.3–21.5	0.001
Consumption of BLT (bacon, lettuce, and tomato)/bacon-containing pre-packaged sandwich	14	32.6	1	1.6	6.7	1.0–43.4	0.047

Model 2: STEC O145 cases with market panel controls							
Exposure	Cases (*n* = 43)		Controls (*n* = 93)		aOR	95% CI	*p* value
	*n*	%	*n*	%			
Age (ref: 11–18 year olds)							
Age: 11–18 year olds	6	14.0	13	14.0	REF		
Age: 19–34 year olds	25	58.1	43	46.2	2.1	0.5–8.4	0.294
Age: 35–70 year olds	12	27.9	37	39.8	1.1	0.3–4.5	0.891
Sex (ref: female)							
Female	28	65.1	58	62.4	REF		
Male	15	34.9	35	37.6	1.0	0.4–2.6	0.969
Consumption of BLT(bacon, lettuce, and tomato)/bacon-containing pre-packaged sandwich	14	32.6	3	3.2	6.4	1.7–24.7	0.007
Consumption of any lettuce leaf in a pre-packaged sandwich	25	58.1	15	16.1	4.8	1.9–12.0	0.001
Consumption of minced beef	15	34.9	18	19.4	2.7	1.0–6.9	0.044

*Abbreviations:* aOR, Adjusted odds ratio; CI, Confidence Interval.

### Comparison of exposures reported in control groups

When looking at the differences between the two control groups, some differences were identified in food exposures.

In single variable analyses, the consumption of avocado (OR: 0.2, 95% CI: 0.0–0.8, *p* = 0.011), red peppers (OR: 0.5, 95% CI: 0.2–1.0, *p* = 0.034), other fruit (OR: 0.5, 95% CI: 0.2–1.0, *p* = 0.038) and chilli sauce in a burger, hotdog or kebab (OR: 0.0, 95% CI: 0.0–1.2, *p* = 0.040) were negatively associated with being a *Salmonella* case–control. The consumption of lettuce (OR: 3.7, 95% CI: 1.0–17.1, *p* = 0.027) was positively associated with being a *Salmonella* case–control.

In the multivariable analysis, accounting for age and sex, being a *Salmonella* control was again negatively associated with consumption of red peppers (aOR: 0.4, 95% CI: 0.2–0.9, *p* = 0.032) and chilli sauce in a burger, hot-dog, or kebab (aOR: <0.1, 95% CI: 0.0–0.9, *p* = 0.043). The consumption of mixed leaves other than those specifically asked about (aOR: 4.7, 95% CI: 1.4–16.2, *p* = 0.013) and chicken that was eaten hot (aOR: 2.2, 95% CI: 1.1–4.5, *p* = 0.031) were now positively associated with being a *Salmonella* control (Table 2).

**Table 2.** Multivariable analysis comparing Salmonella case–controls to MPCs Table 2. long description.

Exposure	aOR	95% CI	*p* value
Age (ref: 11–18 year olds)			
Age: 19–29 year olds	**1.9**	[0.6–5.7]	0.275
Age: 30–70 year old	**0.7**	[0.3–1.8]	0.436
Sex (ref: female)			
Male	**0.6**	[0.3–1.3]	0.222
Avocado	**0.2**	[0.1–1.0]	0.053
Red peppers	**0.4**	[0.2–0.9]	**0.032**
Other fruit	**0.4**	[0.2–1.0]	0.051
Chilli sauce in a burger/hotdog/kebab	**<0.1**	[0.0–0.9]	**0.043**
Other mixed leaves	**4.7**	[1.4–16.2]	**0.013**
Hot chicken	**2.2**	[1.1–4.5]	**0.031**

Results based on single variable results where *p* < 0.1. Bold value signifies p value less than 0.05.

*Abbreviations:* aOR, Adjusted odds ratio; CI, Confidence Interval.

### FDR procedure

There were a total of 103 exposures, of which four were conventionally associated with the control group in the single variable analysis (i.e., p values below 0.05). Given the large number of comparisons being made it is likely that some of these may be falsely associated; that is, there is no true difference in these exposures in the populations from which these two control sets were selected. By conducting Benjamini–Hochberg FDR procedure on the p values from the single variable analysis, there were no exposures with a q value (the FDR-adjusted p value) that were significantly associated with being a *Salmonella* control or MPC at the *q* < 0.1 significance threshold.

## Discussion

There are many challenges faced by OCTs in the selection of a representative set of controls from those in the population at risk. Market research panels are becoming more frequently used in outbreak investigations of gastrointestinal disease, although it is still not routine. However, those participating in panels are likely to have some differences in lifestyle compared to the general population, which could result in biased comparisons. In this analysis, we have described two contemporaneous case–control studies undertaken in response to an outbreak of STEC O145 in the UK using two different control groups – MPCs and *Salmonella* cases. Both studies identified the same food product (pre-packaged sandwiches containing salad leaves) as being the exposure most strongly associated with being a case of STEC O145 in this outbreak [5]. Fresh produce, including salad leaves, has emerged in the UK in the last 20 years as a common vehicle for infection in large, national foodborne outbreaks. In such outbreaks, it is vital that the rapid identification of the source of infection occurs to allow traceback of the product, as the shelf life of such products is short [2–4, 9, 11].

Our experience in this outbreak investigation aligns with that of others [7, 9] in terms of cost saving and time to organise with the market research panel company, recruit controls, and collect the data. This study was more costly per control than the previously reported investigations that used MPCs between 2013 and 2016 (range: £2.00–£3.60 per control). This could be a result of inflation and the cost-of-living crisis, where prices of services have outpaced inflation [7, 9]. Despite the increased cost of MPCs, the cost of using traditional epidemiology methods of control recruitment, using cases of *Salmonella* as controls, was over five times greater than the use of online market panel members in this outbreak. However, recruiting controls by telephone is naturally more expensive because it requires staff time, multiple call attempts, and manual data collection, whereas MPCs are self-completed online at a low marginal cost. Additionally, in this outbreak, the staff time utilised to select, recruit, and complete the survey using the *Salmonella* cases was almost nine times greater than the staff time required to recruit market panel members. Market research panels allow for easy frequency matching of controls, which took additional time to calculate and identify within the recruitment of *Salmonella* case–controls. However, despite a delay in response time from the market control panel provider, the timeliness of the two studies was similar, with MPCs taking two working days from starting to contact controls to closing recruitment, and Salmonella cases taking three working days to recruit. The 2 days taken to organise with the market research company to the point of survey distribution were quicker than the median of 5 days described by Mook et al., which reviewed a number of outbreaks using market research panels (range: 2–18 days) [7]. Delays associated with the MPC provider increased the time from the initial email sent to the provider to the closure of recruitment. However, following the establishment of communication with the company provider, the survey was live and sent to individuals in each strata in just 7 hours.

There were no statistical differences between *Salmonella* case–controls and MPCs in terms of age, sex, and country of residence. In terms of exposures, differences were seen between MPCs and *Salmonella* case–controls, but it would be expected to see random differences, and these were not important. *Salmonella* case–controls were more likely than MPCs to have consumed cooked chicken that was hot – this may reflect the source of these individuals’ *Salmonella* infection, as chicken is a known risk factor for *Salmonella* in the UK and more widely [12]. *Salmonella* case–controls were also more likely than MPCs to have consumed mixed leaves. This may again reflect the acquisition of *Salmonella* in the UK, where in recent years, a number of fresh produce items have been associated with outbreaks [13, 14].

There are limitations to this study. Consistently across both groups, there were challenges with the recruitment of individuals in the 19- to 34-year-old age stratum. For the *Salmonella* case–control groups, this may reflect an increasing issue with individuals choosing not to answer calls from unknown numbers, or, for both groups, it may reflect unavailability due to the time of day at which controls were contacted (i.e., between 9 am and 5 pm, from Mondays to Fridays). In addition, some staff costs and time were estimated; for example, the start of the pay band for the staff members’ grade was used for the calculations, as specific salaries were unknown. The directions of estimates were conservative, hence would underestimate the costs of staff using *Salmonella* case–controls. The questionnaire was distributed in English only to the MPCs; therefore, the survey responses are limited to individuals who can read English. This limitation also applies to the recruitment of the *Salmonella* case–controls; however, for the *Salmonella* case–controls resident in Wales, the survey was translated into Welsh, as English may not have been an individual’s first language. Additionally, there was a longer recall period for *Salmonella* case–controls, who reported food history for the 7 days prior to illness, compared to the MPCs, who reported the 7 days prior to survey completion. This difference in recall period may have introduced differential recall bias between the control groups. In addition, differences in ethnic group and social class between the control groups were not analysed as this information was not collected. The decision not to collect this information was taken as ethnicity and social class were not considered relevant confounding factors in this outbreak. However, the market research company has the ability to collect this information in a future outbreak. It may be that there are limitations to the use of market panels when outbreaks disproportionately affect specific subpopulations or where the implicated vehicle is not commonly consumed across all societal groups.

## Conclusion

From this analysis comparing the differences in exposures reported by controls, the timeliness, and the cost effectiveness of MPCs compared to traditional recruited controls, we would suggest that using MPCs is more time and cost efficient than traditional methods of control recruitment. Delays due to external factors, such as late responses from market panel providers or contractual and administrative processes, should be accounted for when designing studies where possible.

This comparison of two methods of control recruitment, undertaken in parallel during the same outbreak investigation, highlights the benefits of using MPCs in case–control studies investigating dispersed GI outbreaks, particularly those involving fresh produce. Both studies identified the same exposure as being most strongly associated with being a case in the outbreak: pre-packaged sandwiches containing lettuce. This investigation, in conjunction with the food chain investigation, led to a recall of affected products in June 2024 [15], highlighting the importance of rapid epidemiological information during outbreaks involving fresh produce.

## Long descriptions

### Table 1. Long description

The table is divided into two vertical sections: Model 1 and Model 2.

Model 1: S T E C O 145 cases with Salmonella cases as case-controls (n equals 43 cases, n equals 63 controls).

* Age 11 to 18: 6 cases (14.0 percent), 12 controls (19.0 percent). Reference group.

* Age 19 to 29: 25 cases (58.1 percent), 18 controls (28.6 percent). a O R 4.4, 95 percent C I 0.9 to 21.3, p value 0.063.

* Age 30 to 70: 12 cases (27.9 percent), 33 controls (52.4 percent). a O R 2.3, 95 percent C I 0.5 to 10.8, p value 0.300.

* Sex: Female is reference. Male: 15 cases (34.9 percent), 22 controls (34.9 percent). a O R 1.2, 95 percent C I 0.4 to 3.1, p value 0.280.

* Consumption of any lettuce leaf in a pre-packaged sandwich: 25 cases (58.1 percent), 6 controls (9.5 percent). a O R 7.1, 95 percent C I 2.3 to 21.5, p value 0.001.

* Consumption of B L T or bacon-containing pre-packaged sandwich: 14 cases (32.6 percent), 1 control (1.6 percent). a O R 6.7, 95 percent C I 1.0 to 43.4, p value 0.047.

Model 2: S T E C O 145 cases with market panel controls (n equals 43 cases, n equals 93 controls).

* Age 11 to 18: 6 cases (14.0 percent), 13 controls (14.0 percent). Reference group.

* Age 19 to 34: 25 cases (58.1 percent), 43 controls (46.2 percent). a O R 2.1, 95 percent C I 0.5 to 8.4, p value 0.294.

* Age 35 to 70: 12 cases (27.9 percent), 37 controls (39.8 percent). a O R 1.1, 95 percent C I 0.3 to 4.5, p value 0.891.

* Sex: Female is reference. Male: 15 cases (34.9 percent), 35 controls (37.6 percent). a O R 1.0, 95 percent C I 0.4 to 2.6, p value 0.969.

* Consumption of B L T or bacon-containing pre-packaged sandwich: 14 cases (32.6 percent), 3 controls (3.2 percent). a O R 6.4, 95 percent C I 1.7 to 24.7, p value 0.007.

* Consumption of any lettuce leaf in a pre-packaged sandwich: 25 cases (58.1 percent), 15 controls (16.1 percent). a O R 4.8, 95 percent C I 1.9 to 12.0, p value 0.001.

* Consumption of minced beef: 15 cases (34.9 percent), 18 controls (19.4 percent). a O R 2.7, 95 percent C I 1.0 to 6.9, p value 0.044.

Navigate back to Table 1..

### Table 2. Long description

Beginning at the top, the first section lists age exposures with reference group 11 to 18 year olds. Age 19 to 29 year olds has aOR 1.9, 95 percent CI 0.6 to 5.7, p value 0.275. Age 30 to 70 year old has aOR 0.7, 95 percent CI 0.3 to 1.8, p value 0.436. Next, sex exposures reference female. Male has aOR 0.6, 95 percent CI 0.3 to 1.3, p value 0.222. The following rows list food exposures. Avocado has aOR 0.2, 95 percent CI 0.1 to 1.0, p value 0.053. Red peppers show aOR 0.4, 95 percent CI 0.2 to 0.9, p value 0.032. Other fruit has a OR 0.4, 95 percent CI 0.2 to 1.0, p value 0.051. Chilli sauce in a burger, hotdog, or kebab has aOR less than 0.1, 95 percent CI 0.0 to 0.9, p value 0.043. Other mixed leaves have aOR 4.7, 95 percent CI 1.4 to 16.2, p value 0.013. Hot chicken has aOR 2.2, 95 percent CI 1.1 to 4.5, p value 0.031. Significant p values are bolded for red peppers, chilli sauce, other mixed leaves, and hot chicken.

Navigate back to Table 2..
